# Artificial selection for timing of dispersal in predatory mites yields lines that differ in prey exploitation strategies

**DOI:** 10.1002/ece3.8760

**Published:** 2022-03-22

**Authors:** Alexandra M. Revynthi, Dirk Verkleij, Arne Janssen, Martijn Egas

**Affiliations:** ^1^ Institute of Biodiversity and Ecosystem Dynamics University of Amsterdam Amsterdam The Netherlands; ^2^ Department of Entomology and Nematology Tropical Research and Education Center University of Florida Homestead Florida USA; ^3^ Department of Entomology Federal University of Viçosa Viçosa Brazil

**Keywords:** aerial dispersal, Killer, local dynamics, metapopulation dynamics, Milker, *Phytoseiulus persimilis*, spider mites

## Abstract

Dispersal is the main determinant of the dynamics and persistence of predator–prey metapopulations. When defining dispersal as a predator exploitation strategy, theory predicts the existence of a continuum of strategies: from some dispersal throughout the predator–prey interaction (the Milker strategy) to dispersal only after the prey had been exterminated (the Killer strategy). These dispersal strategies relate to differences in prey exploitation at the population level, with more dispersal leading to longer predator–prey interaction times and higher cumulative numbers of dispersing predators. In the predatory mite *Phytoseiulus persimilis*, empirical studies have shown genetic variation for prey exploitation as well as for the timing of aerial dispersal in the presence of prey. Here, we test whether artificial selection for lines that differ in timing of dispersal also results in these lines differing in prey exploitation. Six rounds of selection for early or late dispersal resulted in predator lines displaying earlier or later dispersal. Moreover, it resulted—at the population level—in predicted differences in the local predator–prey interaction time and in the cumulative numbers of dispersers in a population dynamics experiment. We pose that timing of dispersal is a heritable trait that can be selected in *P*. *persimilis*, which results in lines that show quantitative differences in local predator–prey dynamics. This opens ways to experimentally investigate the evolution of alternative prey exploitation strategies and to select for predator strains with prey exploitation strategies resulting in better biological control.

## Graphical abstract

Theoretical studies predict the existence of a continuum of dispersal strategies: from Milker, an early dispersal strategy, to Killer, a late dispersal strategy. We selected for early and late dispersing *Phytoseiulus persimilis* mites from rose leaves with two‐spotted spider mites as prey. We pose that timing of dispersal is a heritable trait that can be selected, which results in lines with quantitative differences in local predator–prey dynamics.

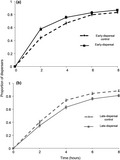

## 1 INTRODUCTION

Dispersal, the movement of individuals from their natal site, is a key process in the persistence of metapopulations and has major consequences for individual fitness, for gene flow among populations, and for population dynamics (Bowler & Benton, 2005, 2009; Clobert et al., 2009; Ellner et al., 2001; Janssen et al., 1997; Revilla et al., 2004; Ronce, 2007; Zemek & Nachman, 1998, 1999). Dispersal connects local populations and allows for colonization of new patches, thus contributing to spatial distributions (Clobert et al., 2009). Even when local populations go extinct, persistence can be observed at a metapopulation level because of the asynchronies of the dynamics in these local populations and the founding of new populations by dispersing individuals (Crowley, 1981; Hilborn, 1975; Jacob et al., 2019; Jansen & Sabelis, 1992; Janssen et al., 1997; Vandermeer, 1973). In fragmented landscapes with many small, individual, isolated patches, dispersal plays a major role in decreasing competition, reducing inbreeding, and providing an escape from adverse biotic and abiotic conditions (Cote et al., 2017; Duputié & Massol, 2013).

Spatial dynamics can be affected by rapid evolution, with current theory suggesting that dispersal rates increase at range margins due to kin competition and spatial selection (Kubisch et al., 2013). At such margins, local populations are isolated and the probability of invasion by competitors is low. In these areas, the exploitation of a growing food source by a consumer population at a rate that maximizes the long‐term yield, also known as prudent predation (Slobodkin, 1968), is therefore evolutionarily stable (cf. Maynard, 1964).

A consumer population can show prudent exploitative behavior either by reducing consumption of the food source or by increasing its dispersal rate. In 1995, van Baalen and Sabelis described the Milker–Killer dilemma, a theoretical framework that describes early dispersal as a form of prudent predation. This dilemma describes the exploitation dynamics in a local consumer population feeding on a reproducing resource population, and distinguishes a continuum of exploitation strategies, ranging from prudent (Milker) to selfish (Killer). A Milker consumer disperses early, after some reproduction, but before the food source is depleted. As a consequence, it does not produce the maximum number of offspring that the food source would allow, so this results in decreased direct fitness of the dispersing individual. However, the dispersal also results in a decrease in the speed with which resources are exploited and the resources left can reproduce, resulting in a longer interaction period (i.e., the time between consumer invasion and food source depletion) between the remaining consumers and the resource. Consequently, the offspring of the dispersed individual that stayed behind on the patch will have more food available, and this prudent exploitation will therefore result in the production of more dispersing individuals during the total interaction period, resulting in a higher inclusive fitness of the dispersed individual. This is especially the case if this increased dispersal is a heritable trait, for example, if the offspring use the same exploitation strategy. In contrast, a Killer individual disperses only after depletion of the food, so produces the maximum number of offspring allowed by the limited food source, and consequently has a high direct fitness. However, its offspring will have a shorter interaction time with the food source and produce fewer dispersing individuals during the entire interaction period, therefore, Killer individuals may have a decreased inclusive fitness. The tradeoff between Milker‐like and Killer‐like strategies seems to be one of life history (Fronhofer & Altermatt, 2015; Kneitel & Chase, 2004). Theory predicts that Milker‐like strategies will be favored only when local predator populations are sufficiently isolated from each other, thus reducing the risk of invasions by Killers, which can benefit from the Milker strategy (Pels et al., 2002; Pels & Sabelis, 1999; van Baalen & Sabelis, 1995) by consuming the resource left behind by the dispersing Milkers.

Here, we investigate whether there is genetic variation in the dispersal strategy in a predatory mite, and whether it is possible to select for either of the two strategies. The acarine predator *Phytoseiulus persimilis* Athias–Henriot (Acari: Phytoseiidae) and its prey, the two‐spotted spider mite (*Tetranychus urticae* Koch; Acari: Tetranychidae), form an ideal system to study theoretical predictions of alternative exploitation strategies. The two‐spotted spider mite naturally occurs in local populations where it can be driven to extinction by its predator *P*. *persimilis* (Jansen & Sabelis, 1992). Upon prey depletion, the predators need to disperse aerially, carried passively by the wind, to find another prey patch. Pels and Sabelis (1999) investigated the dispersal strategies of several field populations of these predators. In agreement with theory (van Baalen & Sabelis, 1995), they found that an isofemale line (i.e., a line originating from a single female) of *P*. *persimilis* from a population that was connected with other populations by dispersal, only dispersed when prey patches were depleted (the Killer strategy). In contrast, an isofemale line of predators from an isolated population dispersed before the prey patch was entirely depleted (Milker). Also, in agreement with theory, dispersal before prey depletion was associated with a longer interaction period (Pels & Sabelis, 1999). In an attempt to repeat this, Revynthi et al. (2018) collected *P*. *persimilis* from six sites along the coast of Turkey and from five sites on Sicily and investigated their dispersal behavior and population dynamics in a set‐up similar to that of Pels and Sabelis (1999). They found large variation in prey exploitation strategies and significant differences in dispersal rates. Together, these studies show that there is variation in behavior regarding prey exploitation and timing of aerial dispersal among lines and populations and suggest that there is a genetic component for dispersal tendency. Yet, evidence of genetically determined alternative aerial dispersal strategies remains absent, and this is what we address here.

Several studies show that dispersal behavior in *P*. *persimilis* and closely related phytoseiid species is, to some extent, genetically determined and heritable (Jia et al., 2002; Maeda, 2005; Nachappa et al., 2009). These studies, however, focused on ambulatory dispersal of this predator rather than dispersal by means of air currents, which is what we studied here. These modes of dispersal are fundamentally different in both the mechanisms that trigger them and their consequences: ambulatory dispersal is triggered by cues that indicate the nearby presence of prey (Mayland et al., 2000), whereas such cues suppress aerial dispersal (Sabelis & Afman, 1994). Moreover, ambulatory dispersal is reversible—the predators can always backtrack—and aerial dispersal, where mites are carried away by the wind, is not, so can have more severe consequences, such as not finding a new prey patch during the rest of the disperser's life. Taking into account that previous research regarding alternative dispersal strategies assumed that aerial dispersal rates have a genetic component (Pels & Sabelis, 1999; Revynthi et al., 2018), we investigated whether it is possible to select for Milker‐like and Killer‐like predatory mite lines in a bidirectional artificial selection experiment, that is, one line selected for early aerial dispersal, the other for late dispersal. The timing of dispersal in these selection lines was compared to control (unselected) lines. Furthermore, to test the theoretical predictions that early timing of dispersal does result in a longer interaction period between prey and predator and a larger number of dispersers from prey patches, we conducted population dynamics experiments using the two selected predator lines.

## 2 MATERIALS AND METHODS

### 2.1 Host plants

Roses (*Rosa* sp. var. Avalanche) were used as a host plant. They are susceptible to spider mites and a single leaf can be maintained fresh in floral foam for more than 3 weeks, which is sufficient time to conduct the experiments described below (see Revynthi et al., 2018). Young rose plants (Olij Rozen, De Kwakel, the Netherlands) were transferred to a climate room, where they were hydroponically grown on rock wool. Conditions in the climate room were 25°C, 70% RH, and 16L:8D. The rose plants were watered two times per week and fertilized (20‐10‐20 N‐P‐K) once per week.

### 2.2 Spider mites

Two‐spotted spider mites (*T*. *urticae*) were originally collected from cucumber plants in a commercial greenhouse in May 1994 (Pallini et al., 1997). The spider mite culture was kept on lima bean plants (*Phaseolus lunatus* L.) in a climate room at 26°C, 50% HR, and 16L:8D.

Cohorts were created to obtain adult females of approximately 2 days into adulthood. One hundred and fifty adult female spider mites were divided over the two primary leaves of a lima bean plant. The leaves were placed on a bed of water‐saturated cotton wool in a plastic tray, which kept them turgid, and prevented the spider mites from dispersing because spider mites cannot walk over wet cotton wool. The females were left on the bean leaves to oviposit for 48 h, after which they were removed, and their offspring were allowed to develop. The cohorts were kept in a climate room at 25°C, 65% RH, and 16L:8D for 17 days.

### 2.3 Predatory mites

**FIGURE 1:**
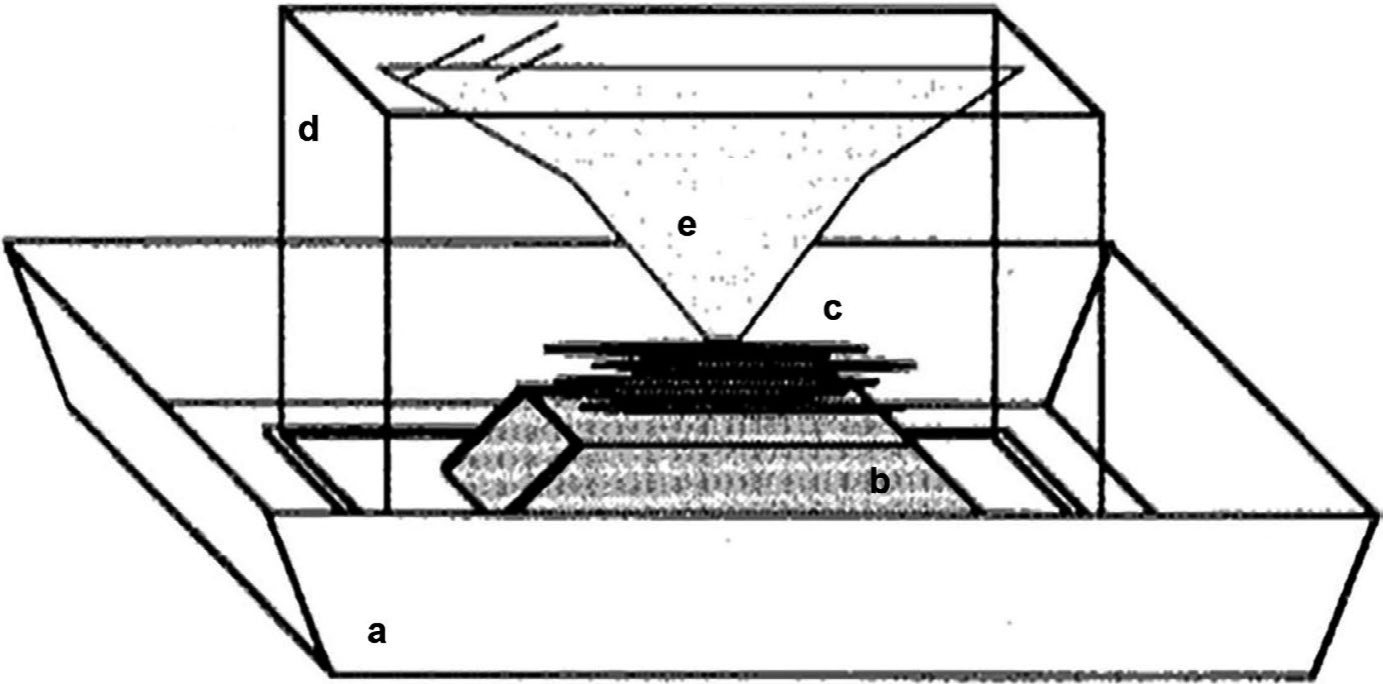
The rearing unit used for predator colonies consisted of (a) a plastic tray filled with soapy water, (b) a plastic float, where (c) infested bean leaves with Two‐spotted spider mites (*Tetranychus urticae*) were placed as a food source for the predators covered by (d) an upside‐down plastic aquarium with (e) a piece of fine‐meshed flexible gauze hanging from its ceiling. The gauze allowed the predators to disperse from the prey patch and subsequently return without drowning, in this way reducing the selection against mites with higher dispersal. Figure modified from Pels and Sabelis (1999)

Six predator strains collected in Turkey in 2013 and five strains collected from Sicily in 2014 were shown to harbor significant variation in dispersal rates, ranging from more Milker‐like to more Killer‐like strategies (Revynthi et al., 2018). On average, 100 predatory mites (both males and females) were used to start the cultures of each of these strains. To establish a base population with sufficient genetic variation for subsequent selection, two gravid females of each of the 11 strains were haphazardly sampled and placed together on lima bean leaflets infested with two‐spotted spider mites. Although we did not explicitly test whether the different predator strains were genetically mixed, we have no reason to doubt it. All collected predators were morphologically and molecularly confirmed as the same species (Revynthi, 2017; Revynthi et al., 2018). This mixed culture was reared for at least 3 months prior to the start of the experiments, with a method described by Pels and Sabelis (1999). In short, bean leaflets were isolated on a plastic float, which was placed in a plastic tray that was filled with a 15 mm layer of water with dissolved soap. To reduce selection against mites with higher ambulatory dispersal tendency, the plastic float was covered with a plastic aquarium (19.5 × 13.0 × 11.5 cm) with a piece of fine‐meshed flexible gauze (80 μm) hanging from the ceiling that connected to the float or leaflets (Figure 1). In this way, the mites had the opportunity to walk from the leaves with prey and consequently return without drowning. This method ensured that the predators that left the leaves with prey did not disappear, hence it reduced the selection for predators that did not disperse in the presence of prey for the hypothetical case that ambulatory and aerial dispersal are correlated behaviors, in which case it would also result in selection for Milkers. Rectangular holes were made in the ceiling of the aquaria and were covered with mite‐proof mesh (80 μm) for ventilation (Figure 1). Individual plastic trays were placed in a fine mesh (80 μm) cage. The cultures were fed three times per week by adding two spider mite‐infested lima bean leaflets to the floats and were kept in a climate room at 25°C, 70% RH, and 16L:8D.

To obtain sufficient numbers of gravid females of the same age (2 days into adulthood) for selection and experiments, cohorts were created as follows. Ten gravid female predatory mites from the base population were placed on a spider mite‐infested bean leaf on a bed of water‐saturated cotton wool in a Petri dish (14 cm diameter × 2 cm). In this way, the leaves remained turgid for at least 10 days. The gravid females were allowed to oviposit for 48 h, after which they were removed and only their eggs and prey were left on the leaves. The cohorts were kept in a climate room at 25°C, 70% RH, and 16L:8D for 10 days.

Two‐spotted spider mites can reach adulthood within 12 days (Laing, 1969) and *P*. *persimilis* within 6 days, both at 25°C (Laing, 1968; Sabelis, 1981). Adult *P*. *persimilis* can prey on all stages of their prey but prefer to feed on eggs and immatures (Takafuji & Chant, 1976), and larvae of this predator do not feed (Laing, 1968; Sabelis, 1981). Adult predator females lay four to six eggs per day on average and need to consume at least six spider mite eggs to produce one egg of their own (Laing, 1968; Sabelis, 1981).

### 2.4 Selection procedure

For the selection procedure, nine wind tunnels were prepared as in Revynthi et al. (2018) for each round of selection. Each wind tunnel consisted of a plastic aquarium (25.3 × 15.8 × 15.5 cm) with holes (11.5 cm diameter) on both sides, covered with a fine mesh (80 μm). A fan was placed outside the tunnel facing the mesh on one side, which created a constant airflow inside the wind tunnel, which was kept at approximately 0.4 m/s during the selection. The shoots of two rose leaves, each with five leaflets, were inserted in a plastic vial (24.5 mm diameter × 40 mm height) filled with water‐saturated Oasis floral foam and the vial was placed at the upwind end of the wind tunnel. The width and length of the leaves ranged from 57 and 8 to 10 cm, respectively. Each of the rose leaves was infested with ten adult female spider mites 24 h before selection started. At the downwind side of the wind tunnel, a trap was placed in order to capture the aerially dispersing predatory mites. The trap consisted of a Petri dish containing the three top leaflets of a rose leaf with spider mites, with the shoot (ca. 3 cm) inserted through a hole in the lid of an Eppendorf^®^ tube (1.5 ml) filled with wet Oasis^®^ floral foam.

#### 2.4.1 Early‐dispersal line and control

For the first round of selection for early dispersal, 60 gravid predator females were transferred from the cohorts to upwind rose leaves in each of six wind tunnels (replicates). At 2, 4, 6, 8, and 24 h, the trap leaves were replaced with new ones. The dispersed mites on the trap leaves were collected and counted at each time step. Experiments were terminated once the first 20–25 dispersers of each of the six wind tunnels (i.e., 120–150 individuals in total) had been collected. These mites were used to set up a culture of the early‐dispersal line. The remaining 210–240 individuals from the six wind tunnels were discarded. Simultaneously, another 60 gravid predator females were transferred to upwind rose leaves in three other wind tunnels. Using the same time intervals, all dispersed predatory mites were collected, transferred into a common cage and 120 of them were randomly chosen at the end of the 24 h and used to start a control line. We opted for the collection of dispersed individuals instead of those that stayed behind to ensure that all individuals were capable of dispersal and not physically impaired. Because the limited food on the upwind rose leaves was consumed during this selection procedure, all predators dispersed eventually, except for a few that appeared physically impaired. Both lines were kept in rearing units as described above. Subsequently, cohorts were created using gravid females from the selection line and control line seven days after the selection, and the adults from these cohorts were subjected to a new round of selection. The total procedure was repeated for six rounds.

#### 2.4.2 Late‐dispersal line and control

Instead of selecting for predators that did not disperse and running the risk that this would include sick or otherwise disabled mites, we selected for late dispersers in a similar manner as above, except that the 20–25 predators that had dispersed last (within 24 h) were used to set up this line. As above, predators that dispersed 2, 4, 6, and 8 h from the start of the experiment were collected and counted by replacing the traps but were subsequently discarded. A separate control line was started simultaneously in three separate wind tunnels as above. This selection procedure was also repeated for six rounds.

For logistic reasons, the selection of the two lines could not be run simultaneously and maintenance of separate replicates from each selection and control line was not possible. Moreover, a period of 14 days between selection rounds of each line was required to obtain a new generation of adult offspring. Therefore, the selection of the early‐dispersal line and its control were alternated with the late‐dispersal line and its control, and one line of each selection regime was created.

### 2.5 Selection response experiment

To test the effects of selection on dispersal rates, we performed an experiment using a set‐up similar to that of the selection procedure. To standardize the quality of the prey patch from which the predators dispersed, these only contained spider mite web and eggs but no other prey stages. Furthermore, the patch consisted of one rose leaf only but was otherwise similar to those in the selection procedure. Prior to the experiment, 15 2‐day‐old adult female spider mites were allowed to oviposit for 48 h on this leaf, were then removed and the number of spider mite eggs was reduced to 80 per leaf, which was subsequently used for the experiment. A trap was placed at the downwind side of the wind tunnel, as described above (*selection procedure*).

At the start of the experiment, 2 weeks after the end of the selection process, 30 gravid 2‐day‐old female predators from one of the selection lines or its respective control line were placed on the prey patch. The mites that had dispersed to the traps were counted during 8 h with 2 h intervals (as in the selection procedure). In the end, the predators remaining on the prey patch were counted. This experiment was performed in three blocks of four replicates of each selection line and its respective control line. The response to early‐dispersal selection and late‐dispersal selection was measured in separate blocks.

Differences in time to dispersal between the various lines were tested with a time‐to‐event analysis with a Cox proportional hazard model. No predators died during the experiments, and censoring was applied to predators that did not disperse during this time (8 h). The packages survival (Therneau, 2020) and coxme (Therneau, 2015) of the open‐source program R, version 4.1.2 (R Development Core Team, 2021) were used. The selection regime was used as a fixed factor and block (replicate of the experiment) as a random factor. Contrasts were assessed through joining nonsignificant factor levels (Crawley, 2007).

### 2.6 Population dynamics experiment

To measure dispersal of the selected lines in a population‐dynamical context and explicitly test whether selection for early or late dispersal resulted in different exploitation strategies, eight wind tunnels were prepared as in the *selection procedure*. The experimental procedure was the same as in Revynthi et al. (2018): eight rose leaves were infested with 15 2‐day‐old adult female spider mites, each placed in a wind tunnel. These spider mites were allowed to oviposit for 48 h, after which one gravid 2‐day‐old adult female predatory mite was released on each leaf. Subsequently, the adult spider mites could continue ovipositing, the adult female predator could feed on prey eggs, immatures, and adults and reproduce. Adding only one gravid female to each leaf allowed measuring predator dispersal behavior from a single founder and her offspring. Starting on the day on which the predator was added, adult prey on the leaf and predators both on the leaf and dispersed were counted every 24 h until there were no more prey or predators present on the leaf (approx. 20 days after the release of the female predator). Prey mites on the trap were not counted. This experiment was performed in two blocks of four replicates for each selection line, 2 months after the sixth selection round.

Theory predicts that the interaction period of the prey with the predator and the cumulative number of dispersing predators should differ as a consequence of the dispersal rate (van Baalen & Sabelis, 1995). We therefore tested differences in dispersal rate, interaction period, and the cumulative number of dispersers between selected lines. The dispersal rate was calculated per day as the proportion of all predators that had left the arena during that day. Because all predators were expected to disperse when prey was exterminated, we used dispersal rates until the last day that at least 3 adult prey were still present on the patch (Revynthi et al., 2018). These daily dispersal rates were subsequently averaged per replicate and these averages were analyzed (hence, 1 value per replicate). The interaction period was taken as the time interval between the predator introduction on the leaf (day 3 of the experiment) and the time of prey elimination. Because these three response variables were all assessed on the same replicates, a multivariate analysis of variance (MANOVA) was first applied to the dispersal rate, interaction period, and the total number of dispersed predators combined, with selection regime and block as explanatory variables. After having found a significant difference between selected lines with the MANOVA, each response variable was tested separately as follows. There proved to be no significant difference between blocks so we could use simple generalized linear models with a quasi‐Poisson error distribution (log link) for the total numbers of dispersed predators and a Gaussian error distribution (identity link) for the dispersal rates. The interaction period was tested with a time‐to‐event analysis with a Cox proportional hazard model.

## 3 RESULTS

### 3.1 Selection response experiment

**FIGURE 2:**
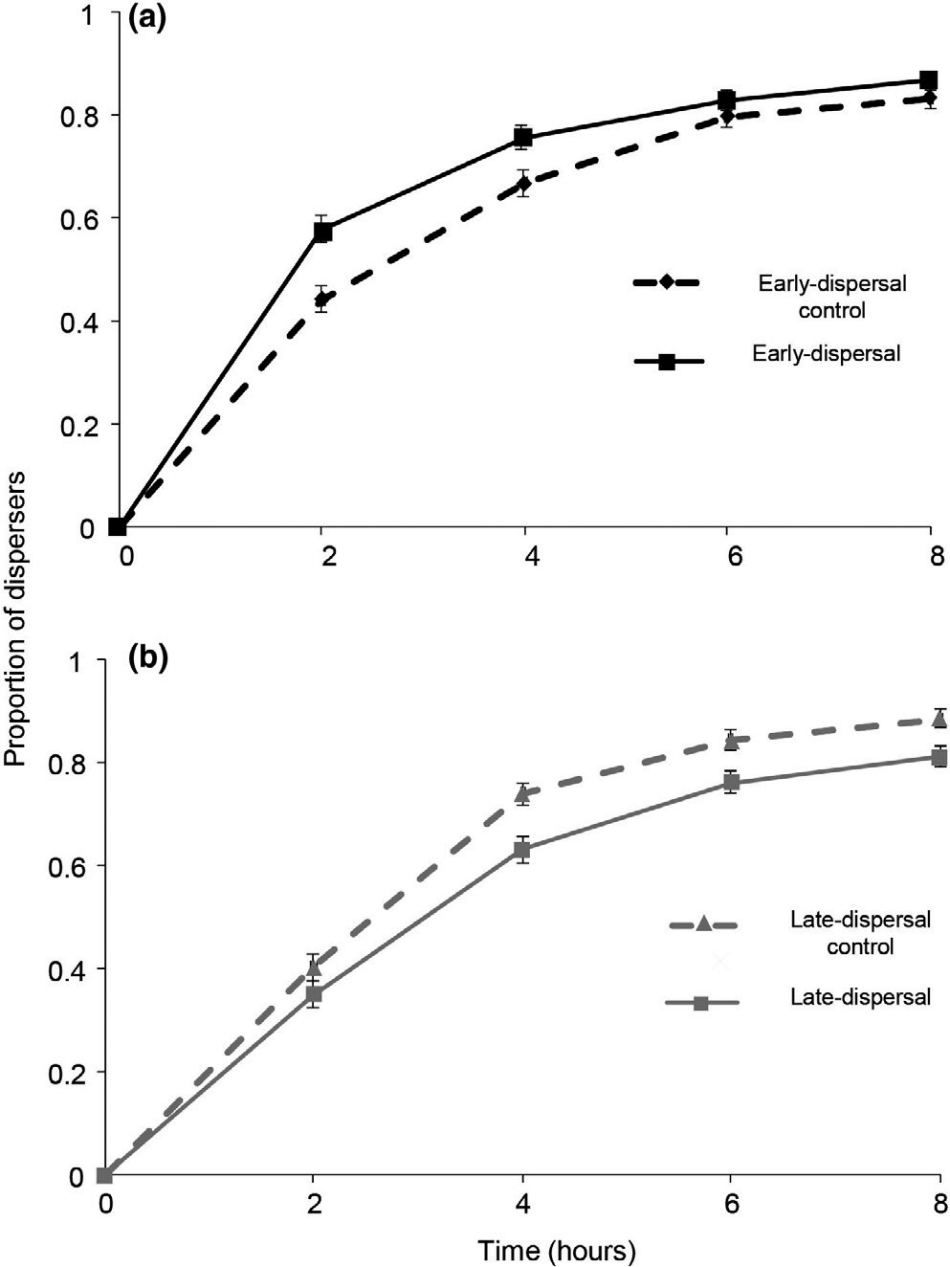
Predatory mites respond to artificial selection on the timing of dispersal. Panels show average proportions of dispersers (±SE) through time. (a) The early‐dispersal selection line and its control line. (b) The late‐dispersal selection line and its control line. Black dashed line with rhombus: early‐dispersal‐control line, black solid line with a square: early‐dispersal line, gray dashed line with triangle: late‐dispersal‐control line, gray solid line with a circle: late‐dispersal line. *N* = 12 for each line

The predators from the early‐dispersal line dispersed significantly earlier than the predators from their control line (Figure 2a, Cox mixed‐effects proportional hazards: χ^2^ = 7.5, df = 1, *p* = .006). Similarly, the predators from the late‐dispersal line stayed significantly longer on the leaf than the predators from their control line (Figure 2b, Cox mixed‐effects proportional hazards: χ^2^ = 8.65, df = 1, *p* = .003). These findings show that there is indeed a heritable component in the dispersal behavior of *P*. *persimilis*.

The two selection procedures were not performed exactly at the same time but in alternating weeks (see *Selection procedure* in Materials and methods). Nevertheless, they were done under identical conditions in the same period, so we are convinced that the results were not affected by this slight asynchrony, hence, we also compared the two selection lines and the two control lines. The predators from the early‐dispersal selection line dispersed significantly earlier than the late‐dispersal selection line (Cox mixed‐effects proportional hazards: *χ* ^2^ = 17.17, df = 1, *p* << .001), and the two control lines did not differ significantly from each other (Cox mixed‐effects proportional hazards: *χ* ^2^ = 1.51, df = 1, *p* = .22).

### 3.2 Population dynamics experiment

**FIGURE 3:**
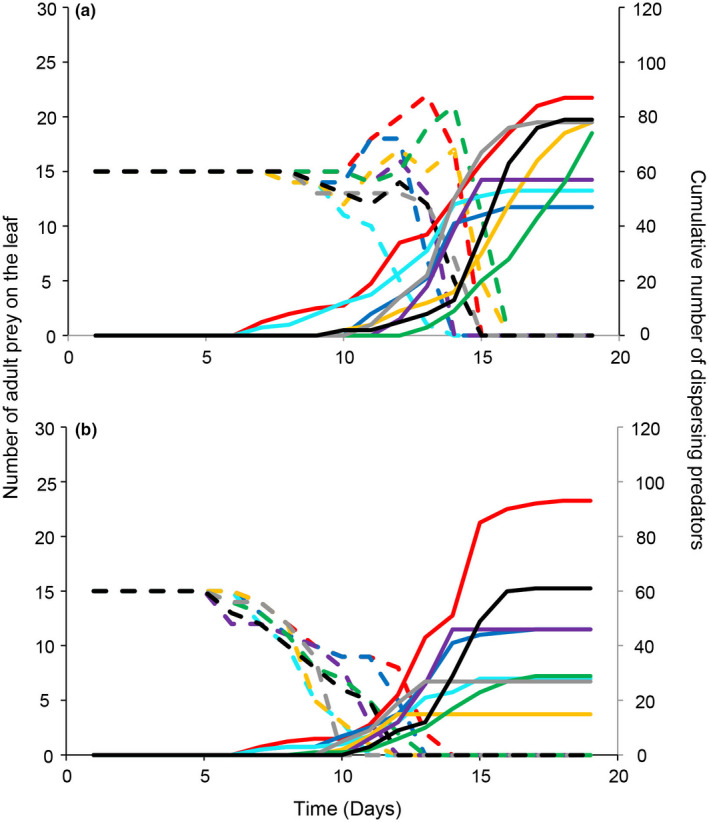
Population dynamics of adult prey on the experimental leaf (dashed lines, left‐hand vertical axis) and a cumulative number of dispersing predators (solid lines, right‐hand vertical axis). (a) Early‐dispersal selection line; (b) Late‐dispersal selection line. Different colors correspond to different replicates. *N* = 8 for each line

We subsequently measured predator dispersal in a population‐dynamical context. Predator–prey population dynamics differed between the two lines (Figure 3). The adult prey population in the early‐dispersal line treatment started decreasing on day 8 (Figure 3a), while in the late‐dispersal line this happened on day 5 (Figure 3b). As expected, predators from the early‐dispersal line initiated dispersal when there were still many adult prey on the leaf in comparison with the late‐dispersal line, which initiated dispersal when the prey population was close to elimination (Figure 3). Differences were also evident in the prey population dynamics. In the experiments with the late‐dispersal line, the adult prey population remained stable for 5 days, after which it decreased monotonously (Figure 3). With the early‐dispersal line, the adult prey population remained stable for approximately 7 days and showed an increase 2 weeks after the start of the experiment (Figure 3), which is approximately the generation time of the prey, indicating that prey offspring had made it to adulthood despite predation.

**FIGURE 4:**
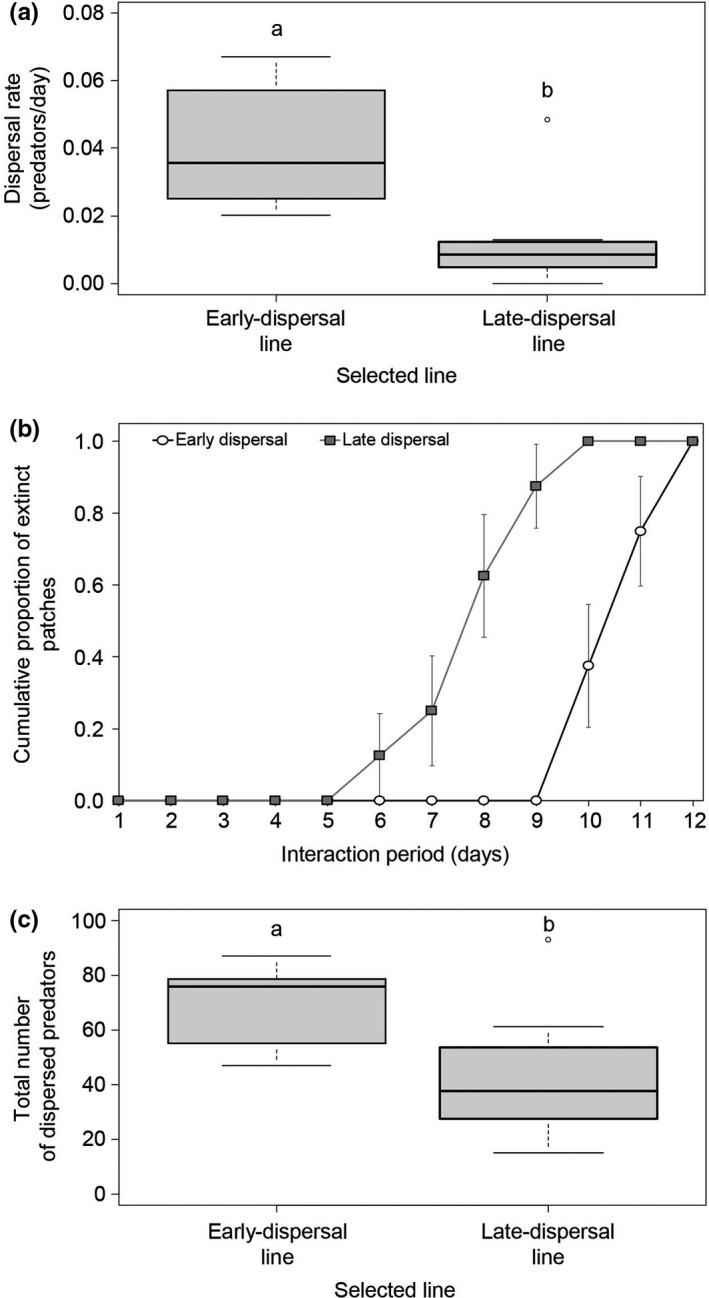
Parameters from the two selected lines in a population dynamics experiment. (a) the dispersal rate during prey exploitation (i.e., the time interval between predator introduction and the last day with at least three adult prey individuals on the leaf), (b) the cumulative proportion of patches that went extinct during the interaction period (i.e., the time interval between predator introduction to the leaf and prey elimination), and (c) the total number of dispersers throughout the experiment. Boxes indicate the second and the third quartile; horizontal lines indicate the medians, whiskers above and below the box indicate the 90th and 10th percentiles. Different letters indicate significant differences (*N* = 8 for each line). Early‐dispersal line: black line with white circles; late‐dispersal line: gray line with gray squares

The combination of the three response variables (dispersal rates during the interaction period, interaction periods, and cumulative numbers of dispersing predators) varied significantly between the selected lines, but not between blocks (MANOVA, Line: *F*_1,3_ = 17.0, *p* < .001, Block: *F*_1,3_ = 1.26, *p* = .34). Subsequent analysis of each response variable separately showed that dispersal rates varied significantly between the selected lines (Figure 4a and S1A, GLM: *F*_1,14_ = 11.0, *p* = .005). In addition, the early‐dispersal line interacted significantly longer with the prey (Figure 4b and S1B, Cox proportional hazards: Likelihood ratio test =12.5, df =1, *p* = .0004) and produced significantly more dispersers than the late‐dispersal line (Figure 4c and S1C, GLM: *F*_1,14_ = 5.61, *p* = .033). These results show that it is possible to select for Milker‐like and Killer‐like predatory mite lines.

## 4 DISCUSSION

We show that it is possible to select for early and late aerial dispersal of the predatory mite *P*. *persimilis*, which supports the hypothesis that there is a genetic basis for the tendency to disperse aerially in *P*. *persimilis*. The population dynamics experiment shows that dispersal rates are intimately connected to patch exploitation strategies. Early predator dispersal (i.e., the so‐called Milker strategy) from a prey patch resulted in prolonged predator–prey interactions on the patch and a higher total number of dispersing predators over the entire interaction period, while late predator dispersal (i.e., the so‐called Killer strategy) resulted in a shorter interaction period and a lower number of dispersing predators. Hence, we selected predators that differed in their timing of dispersal and showed that this resulted in the predicted differences in dynamics on the prey patch. Although this seems logical in hindsight, it is not obvious that selection for early dispersal would result in more prudent patch exploitation. Alternatively, selection for early dispersal could have resulted in selection for individuals that wastefully kill prey, which would not result in prudent exploitation. It is known that *P*. *persimilis* partially ingests prey at high prey densities (Sabelis, 1981, 1985, 1986), which could be viewed as a form of wasteful killing, hence, our selection for early dispersal could have resulted in selection for partial prey ingestion, resulting in earlier extermination of the prey and not in prudent exploitation.

The differences in the adult prey densities between the two lines, with a monotonous decline with the late‐dispersal line but an increase in densities with the early‐dispersal line (Figure 3) might have been due to factors other than predator dispersal. Specifically, predators from the early‐dispersal line might have a lower total population growth rate (counting dispersers and nondispersers) and a lower total predation rate and would therefore have needed more time to deplete the prey eggs and immatures. These delays in predator population growth, as well as the predation rate, may have led to the increase of the adult prey population. Similarly, predators from the late‐dispersal line might have a higher growth rate and predation rate, resulting in faster prey depletion. These differences could have arisen as an unexpected byproduct of the selection procedure. For instance, strong selection in sexually reproducing organisms may increase inbreeding, which can result in inbreeding depression (see Kawecki et al., 2012 for review) that may have expressed itself here in lower growth rate and predation rate. However, the selection of both lines was similar, hence, there is no reason to suspect that selection led to the fixation of deleterious alleles for one of the lines but not the other. Furthermore, if anything, the total numbers of predators and the instantaneous population growth rate of the early‐dispersal line were higher, not lower, than that of the late‐dispersal line during the experiments (Figure S2), showing that there was no effective selection on a decreased growth rate in the former.

Tradeoffs involving dispersal and foraging (Fronhofer & Altermatt, 2015; Kneitel & Chase, 2004) or between attack rates and the conversion of prey into predator biomass (Gibert & Yeakel, 2019) can also explain the differences in the adult prey exploitation of the two lines. Individuals may tradeoff their ability to disperse and find new patches with the ability to overexploit their local food source, for example in rodents (Kotler & Brown, 2003), freshwater snails (Chase et al., 2001), flies (Sokolowski, 1980), and microbes (Fredrickson & Stephanopoulos, 1981). Hence, an alternative explanation for the observed differences in exploitation patterns is that predators from the early‐dispersal line had a higher prey‐to‐predator conversion rate than predators from the late‐dispersal line. Higher prey‐to‐predator conversion rates can lead to higher predator densities without exhausting the prey population. This may be an additional mechanism resulting in Milker‐like exploitation patterns.

Spider mites also overexploit their food source and disperse after that (Sabelis et al., 2002). Although the rose leaf was maintained fresh through the moist floral foam, it was never replaced. This means that the prey food source was limited and of declining quality throughout the experiment. While dispersal of the prey was not observed during the experiments, it is possible that prey individuals dispersed due to plant deterioration or as an antipredator response to avoid predation (Jacobsen et al., 2016). However, the prey used were not selected for differences in exploitation or dispersal strategies, so it is unlikely that the observed differences in dynamics (Figure 3) were caused by differential prey dispersal. It would be interesting, though, to study the joint effects of different prey and predator dispersal and exploitation strategies. For example, the presence of predators may cause selection for early dispersal in the prey to escape from predation. The reduced prey availability, in turn, would select for more Killer‐like predators because the indirect benefit of leaving prey for kin (offspring) is lower. In contrast, overexploitation of the plant by the herbivores sets an upper limit to the predator–prey interaction period, implying that prey exploitation by Milker predators cannot go on indefinitely, thus also setting an upper limit to the Milker‐like dispersal rate.

The predators used in this study were derived from populations with large variation in their dispersal tendency (Revynthi et al., 2018), which were brought to the laboratory at least nine months prior to the start of the experiments. Thus, we tried to control for environmental and maternal effects. Besides being heritable to some extent (Saastamoinen et al., 2018; Stevens et al., 2013, 2014), dispersal behavior can also be highly plastic (Baines et al., 2020; Bitume et al., 2013, 2014; Bonte & Dahirel, 2017; Clobert et al., 2009; Fronhofer et al., 2018; Little et al., 2019), and can vary (Fronhofer et al., 2018; Little et al., 2019). The initial conditions in our population dynamics experiments may have favored prudent predation because a single foundress could exploit the prey patch in her own interest without the existing possibility of invasion by predators with different exploitation strategies (van Baalen & Sabelis, 1995; Pels et al., 2002; Pels & Sabelis, 1999). Because our selected lines show consistent differences in dispersal behavior and prey exploitation under similar initial conditions, our results show that there is an important genetic component involved in the evolution of alternative exploitation strategies of *P*. *persimiilis*. Future experiments that explore whether or how predator dispersal behavior is affected by an invading predator with a different exploitation strategy might yield more in‐depth information on the evolution of alternative exploitation strategies.

Dispersal is also known to vary with the condition and gender of the dispersing individuals (Baines et al., 2020; Bowler & Benton, 2009; Li & Kokko, 2019). For example, females have higher energy requirements than males, because reproduction is costly (Harshman & Zera, 2007) and we can therefore expect that females and males might have different exploitation strategies. Indeed, a previous study has shown that females of *P*. *persimilis* disperse earlier than males when food is limited (Revynthi et al., 2020). Similar behavior was observed in the current and previous population dynamics experiments (Revynthi et al., 2018). Additionally, experiments testing how the degree of relatedness (kin or nonkin) of the dispersers and the predators on the local population will affect the exploitation strategy of the latter can also provide useful information about the evolution and flexibility of these strategies.

In contrast to earlier studies on dispersal by *P*. *persimilis* (Jia et al., 2002; Maeda, 2005; Nachappa et al., 2009), here, the predators could only disperse using the airflow in the wind tunnel and could not return to the prey patch after they had departed. Moreover, the predators could not perceive the cues of the infested leaf that served as a trap, because it was placed downwind from the prey patch. Our study thereby adds to the literature, showing that aerial dispersal in this species has a genetic basis. Furthermore, this aerial dispersal is important for metapopulation dynamics, whereas ambulatory dispersal only serves to cover short distances. Metapopulation experiments with this predator–prey system indeed showed that unlimited ambulatory dispersal resulted in global extinction, and that metapopulation persistence occurred only when ambulatory dispersal was severely limited (Ellner et al., 2001; Janssen et al., 1997). In the future, a direct comparison of ambulatory and aerial dispersal using the selected lines can give more insight into alternative exploitation strategies and the correlations between these two dispersal modes.

We show that six rounds of selection were enough to create lines with different dispersal strategies. Although no contemporaneous replicate selection lines could be created, the control lines provide evidence that the traits measured are stable over time in the absence of selection. Furthermore, the phenotypes were stable 6 months after the selection process, confirming that such selection can also occur in nature. This is an important step in investigating the evolution of alternative prey exploitation strategies because it suggests genetic heritability of and variation in the innate tendency to disperse aerially in natural populations of *P*. *persimilis*, and it opens ways to investigate the consequences of these exploitation strategies experimentally, either alone or when played against each other.

A better understanding of the genetic basis of traits relating to dispersal tendency and prey exploitation behavior can contribute to our understanding of the evolution of alternative exploitation strategies, but it also has applied value. In the case of natural enemies of pests, it can provide a basis for breeding programs to create strains with desirable traits for effective biological control (Le Hesran et al., 2019; Lirakis & Magalhães, 2019). *Phytoseiulus persimilis* is commercially available as a biological control agent of two‐spotted spider mites, so selecting for strains with desirable traits could improve the efficiency of managing spider mite pests. For instance, for the purpose of biological control of spider mites in ornamental crops, it is essential that predatory mites do not disperse before prey elimination, and we show here that such traits can be selected for.

## CONFLICT OF INTEREST

The authors have no conflict of interest to declare.

## AUTHOR CONTRIBUTIONS

**Alexandra M. Revynthi:** Conceptualization (equal); Formal analysis (equal); Investigation (lead); Methodology (equal); Writing – original draft (lead); Writing – review & editing (lead). **Dirk Verkleij:** Conceptualization (equal); Formal analysis (supporting); Investigation (equal); Methodology (equal); Writing – original draft (supporting); Writing – review & editing (supporting). **Arne Janssen:** Conceptualization (supporting); Formal analysis (equal); Funding acquisition (equal); Investigation (equal); Methodology (supporting); Project administration (equal); Supervision (supporting); Writing – original draft (supporting); Writing – review & editing (equal). **Martijn Egas:** Conceptualization (equal); Formal analysis (supporting); Funding acquisition (equal); Investigation (equal); Methodology (equal); Project administration (equal); Supervision (lead); Writing – original draft (supporting); Writing – review & editing (equal).

## Supporting information

Fig S1‐S2

## Data Availability

Data are published at UvA/AUAS Figshare https://doi.org/10.21942/uva.19322555.v1.
