# Supplementary material for: Artificial selection for timing of dispersal in predatory mites yields lines that differ in prey exploitation strategies
Source: Ecol Evol. 2022 Mar 22;12(3):e8760. doi: 10.1002/ece3.8760 (PMC8939366; doi:10.1002/ece3.8760)
Supplement: Supplementary file 1 — Fig S1‐S2 [file ECE3-12-e8760-s001.zip › ece38760-sup-0001-FigS1-S2.docx]

**Figure S1**. Parameters from the two selected lines in the population dynamics experiment. A: the dispersal rate during prey exploitation (i.e. the time interval between predator introduction to the leaf and last day with at least 3 adult prey individuals on the leaf) per replicate, B: the interaction period (i.e. the time interval between predator introduction to the leaf and prey elimination) per replicate and C: the total number of dispersers during the experiment per replicate. Squares: Early-dispersal selection line; Triangles: Late-dispersal selection line.

**Figure S2.** A) Total number of *Phytoseiulus persimilis* predators in the aquarium (on the leaf and dispersed) and B) the instantaneous population growth rate of the early- and late-dispersal line. Black circles: Early-dispersal selection line; White circles: Late-dispersal selection line.
