# Supplementary figures and images for: Artificial selection for timing of dispersal in predatory mites yields lines that differ in prey exploitation strategies
Source: Ecol Evol. 2022 Mar 22;12(3):e8760. doi: 10.1002/ece3.8760 (PMC8939366; doi:10.1002/ece3.8760)

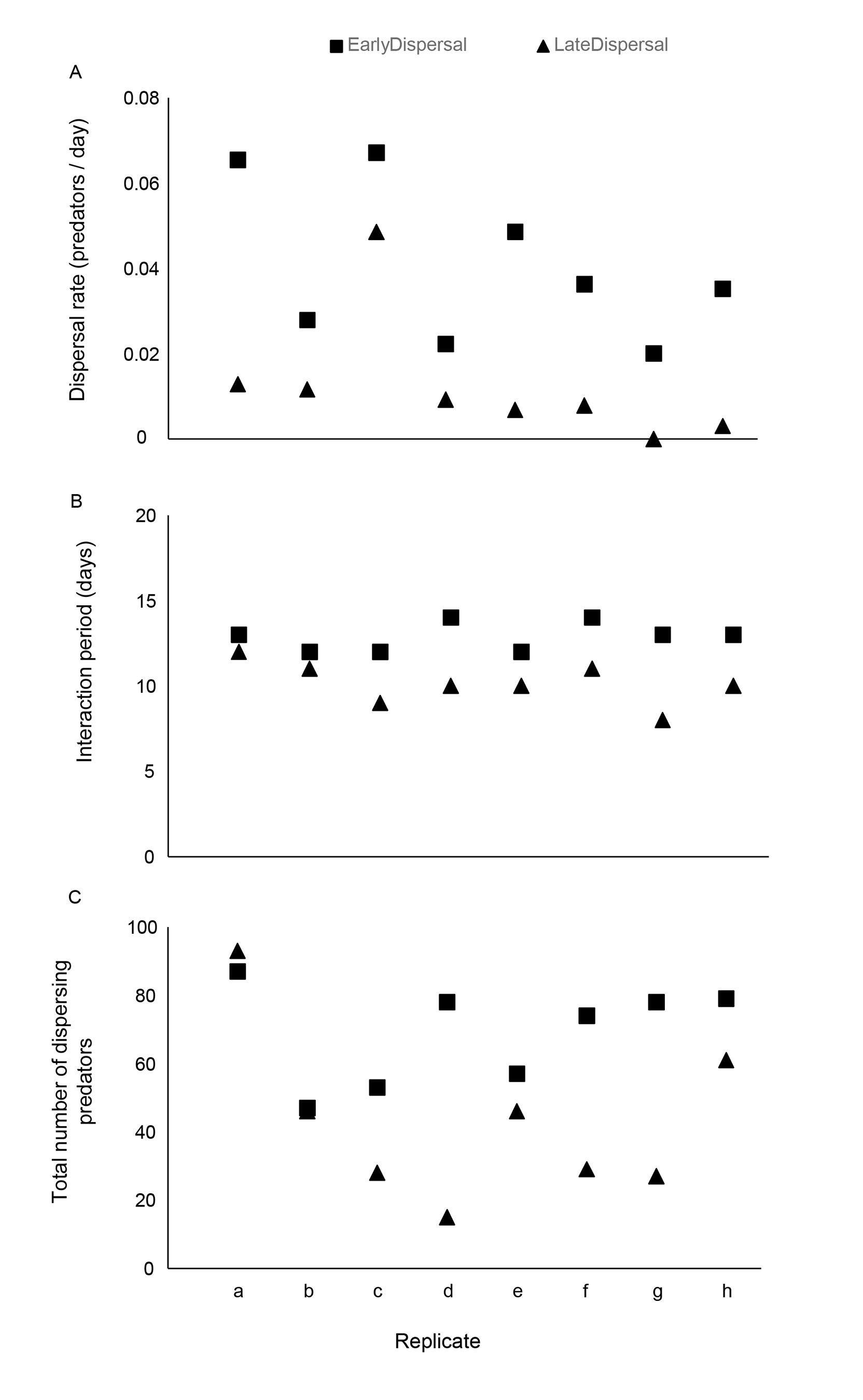

Supplement: Supplementary file 1 — Fig S1‐S2 [file ECE3-12-e8760-s001.zip › ece3_8760_Figure S1.jpg]

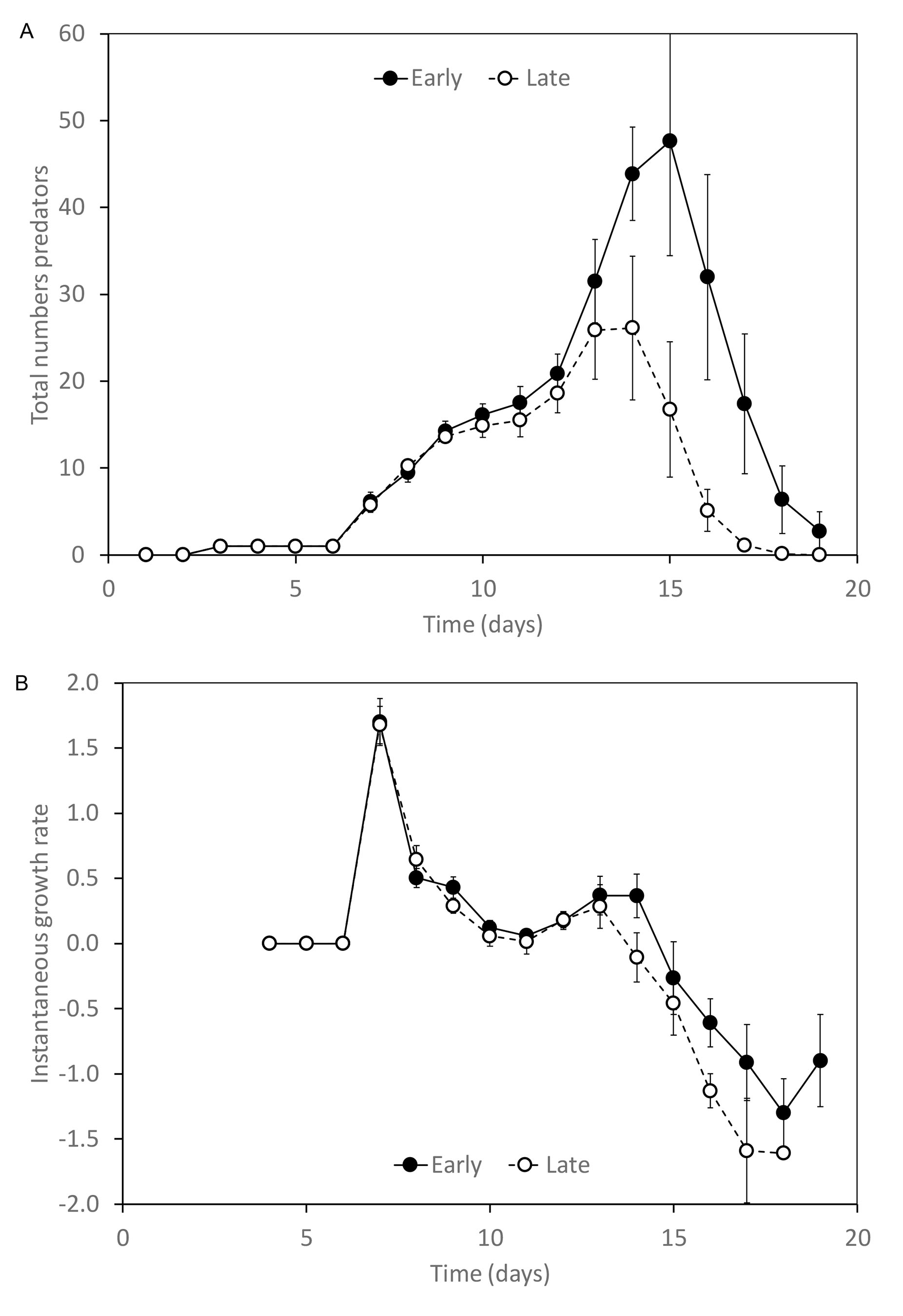

Supplement: Supplementary file 1 — Fig S1‐S2 [file ECE3-12-e8760-s001.zip › ece3_8760_Figure S2.jpg]
